# Therapeutic Notes from My Practice

**Published:** 1887-06

**Authors:** J. S. Todd

**Affiliations:** Professor Materia Medica and Therapeutics, Atlanta Medical College, Atlanta, Ga.


THE ATLANTA MEDICAL AND SURGICAL JOURNAL.

VOL. IV. JUNE, 1887. No. 4.

### Original Communications.

### THERAPEUTIC NOTES FROM MY PRACTICE.

READ BEFORE THE ATLANTA SOCIETY OF MEDICINE, APRIL 26TH, 1887. BY J. S. TODD., M. D., PROFESSOR MATERIA MEDICA AND THERAPEUTICS, ATLANTA MEDICAL COLLEGE, ATLANTA, GA.

QUININE IN INFANTILE COLIC—DIOSCORINE—ERGOT—POLK ROOT—MISTLETOE—ROCKBRIDGE ALUM WATER.

It has been affirmed with truth that the best anecdotes have never been written, neither can they be. Many of our most used medicines in general practice find but small space in the text-books; indeed, like the good jokes, we avoid mention, so great is our fear of the scorpion lash of ridicule held ever threatening up.

We are undoubtedly more scientific than the M. D. of one hundred years ago, but I seriously doubt our being their equals in the art of practicing, prescribing or impressing our patients—mbuing them with the faith in us—that they instilled into their sick. Is it not a fact that, as we become more scientific, we become more skeptical about the action of drugs? Indeed, it is very fashionable and considered smart—I will not say wise—for men, who count themselves better than the average, to say: "I have no confidence in drugs." Of such I can only say, "much learning hath made them mad," and they should be furloughed; or, as a punishment, the old doubter, the skeptic about things in general, past, present and future, but especially things *present*, he should be put in a laboratory and made to study bacteriology.

But without more words, to the subject.

### QUININE IN INFANTILE COLIC.

There is a disease, known among old women as *three-months colic*, that torments infants, and not them alone, unfortunately, for they are none the worse for wear. Would that the same could be said of parents, nurses and neighbors. Such a disease really and truly does exist, but in my reading not in the text-books, or if so it appears under another name. The age at which it develops is generally about the second week after birth. During the day the infant sleeps sweetly and soundly, but as the shades of night deepen the little stranger begins to exercise his lungs, which practice he continues for from two to eight hours. Paregoric, Dewee's carminative, calomel, *et sui generis*, alleviate, palliate somewhat the suffering of the little screamer. The bowels are hard, the legs drawn up, rumblings are heard over the abdomen, and frequently an escape of gas is followed by complete relief of pain. From having made the night up to this time hideous, he relapses into angelic sweetness. We flatter ourselves that the key to the disease has been found, and address ourselves with renewed energy to the digestion, using largely the carminatives, purgatives, etc. Thus we continue day after day, and the child serenely, night after night, continues his serenade. As said before, he is none the worse for wear; he thrives on adversity. No babies are healthier-looking, fatter or so plump. I have had two such children in my own family, or rather one went the full three months despite all and every remedy he took, and five doctors and all the old women tried their hands on him without relief. He could take a drachm of paregoric at a dose when two months old and other narcotics in proportion. He finally wore it out, I reckon; I am sure he did every one else. The other boy started off in the same way and so continued for two weeks, when I concluded, what I ought to have seen before, that the disease being periodical in character, and not attended with fever, was neuralgic, and that I would give him the benefit of the great anti-periodic, quinine. After the first night the neuralgia of the bowels was *cured*, and did not return for fourteen days, when, as by magic, it again vanished under the potent drug. I have used it in every case of this kind since, and with the same unvarying and gratifying success. It might be urged that the disease was malarial, but the absence of fever and the sweating stage and the continual growth of the child I think negatives this view. My friend and colleague, Dr. Taliaferro, tells me that three of his children afflicted him with the affection in question, and resisted all curative agents. I have had in my practice the pleasure of curing about eight cases, all that I have seen, since this mode of procedure was first adopted by me, and so far as I know it is original with me. Dr. James A. Gray has tried this treatment and informs me, with success in every case. I submit these notes for the benefit of both parents and the child. I used the following formula:

℞. Quin. sulph.............................gr. ix.
Hydg. chlo. mit..........................gr. iii.
M. ft. chart.............................. ix.
Sig.—One at 9, 12 and 3 each day.

### DIOSCORINE IN HEPATIC COLIC.

A judge of one of our highest courts was reduced to the verge of the grave with frequent attacks of that excruciating malady, *hepatic colic*. He had the best medical advice that Georgia could give; he vainly sought distant watering-places and other physicians; he was a wreck; gave up his business and lost hope. He was advised to take fluid ext. wild yam—dioscorine—by some one, and never after did he have an attack. I do not mean that one dose cured him, but that from the beginning of its use dated the end of his trouble. He is now one of the finest specimens of the physical man that you meet in our city of healthfulness. He imparted this information to me five years ago. My success with the remedy in hepatic colic has been simply all that I expected or desired. It heightens the pain-relieving power of opium during the attack, and prevents their return when the remedy is continued. I will not worry you with details, but affirm that I have cured cases where parties had suffered for years with oft-repeated attacks. I will not go into the *modus operandi*—that is not the object of this paper. I merely mean to chronicle facts, leaving the explanation of the how to others. Coincidence will be assigned as the reason by many for the curative action I have gotten from the drug. Then all I have to say is that happy are my patients, happy am I, and happier still these strange coincidents that, unlike angels' visits, are *not* few or far between. The Dispensatory (National) gives the drug a few lines only, and sneeringly says: "In Iowa it is known as colic root. . . It may be assumed that it has some of the qualities of ipecac."

FLUID EXTRACT OF ERGOT IN INTERNAL HEMORRHOIDS.

Dr. Jervy, of Virginia, some six years ago, called my attention to the good results that followed the injection of fluid extract ergot into the cavity of the rectum in internal piles. Said some doctor in Richmond, whose name I do not recall, read a paper on the subject, and that he had found it good, etc. Of eight bad cases of internal piles, such as I had formerly immediately advised operative procedure in, *six*, treated with one dr. fluid extract ergot, in same quantity of hot water, injected twice a day, morning and evening, so far recovered that there were no tumors left to excise or tie. The injections were not made in the substance of the tumors, but simply with a p. p. syringe, thrown up and retained in the rectum. In some cases it produced so much uneasiness that it was soon voided. The addition of ten drops of laudanum to each injection secured toleration. This practice cheats the surgeon, but as patient and physician are a majority, his protest in a democratic community will not be considered. Of course this agent will not cure all cases, but six out of eight is a pretty good record.

### POLK ROOT IN MAMMITIS.

POLK ROOT—*Phytolacca decandra*—was advocated by a writer in the *American Journal of Medical Science*, of which I have been a constant reader, more than a decade ago, as a valuable remedy in mammary abscess.

I consider it a true anti-galactagogue. Being such, it rests the inflamed gland, and physiological rest is the greatest of all curative agents in a number of diseases. The surgeon's splints keep at rest the muscles that surround the fractured bone, and also secure their apposition; nature does the knitting; opium is the internal splint that quiets the bowels when they or the peritoneum are inflamed; belladonna paralyzes the muscles of accommodation about the eye, gives it physiological rest and repair uninterrupted, and ever ready does its work.

When I have a case of threatened abscess, and I know it by the pain and swelling of the breast, which are among the very first symptoms, for "*ubi irritatio ibi affluxus*," I give 10 drops of the fluid extract phytolacca decandra and a brisk purgative. Sometimes I empty the breast and put on a bandage; never allow rubbing, handling and poulticing. The dose of phytolacca is repeated every hour for three or four times, then the intervals lengthened gradually *pro re nata.* True I have once failed with this agent, but only once, but I count my successes in averting suppuration by the tens. When it becomes necessary to wean the child, or dry up the milk, or lessen its secretion in those who are weakened by its profuseness, and they are not few, because of the death of the infant or for other reasons, I have found it to be an unfailing and pleasant anti-galactagogue.

### MISTLETOE.

I have but little obstetric practice, but the little that I have has made me yearn for an agent that would increase the natural, rythmical, intermittent pains or contractions of the uterus. Ergot, as you all know, will not do this, the contractions it occasions being non-intermittent, persistent, not remittent, it entails greater suffering on the mother, and enhances the risks of laceration of cervix and perineum, and last, but by no means least, greatly increases the number of still-births.

In a French journal, some four years ago, I read that the agent I desired was found in mistletoe—*viscum flavescens*. I have every year since begged of the students attending my lectures on therapeutics and materia medica in the Atlanta Medical College to use it.

I have half a dozen letters from them, saying it does all the French man of medicine claims for it. I am persuaded that it will fill a long-felt want, and hope that a knowledge of it will become general. [Dr. Green, a member of the society, gives me liberty to say, after the paper had been read, that he had used a decoction of the mistletoe in six cases of tardy labor, and that he was delighted with the results, as it acted to increase only the length and tonicity of the pain, not notably affecting their frequency, and certainly followed by intervals of complete relaxation.]

### ROCKBRIDGE ALUM WATER.

Some four and a half years since I had, among other cases, one of dysentery, which, resisting all medication, became chronic, and a large ulcer of the rectum developed. I called in consultation a noted surgeon. The patient's condition was one of extreme emaciation; blood and mucus in large quantities were voided about an average of once an hour. The dose of opium had been increased to 3 dr. laudanum in twenty-four hours and a ⅓ gr. morphine twice a day, and still matters grew worse. We decided to inject solution 30 grs. to oz. nit. silver in rectum. The result was a collapse that nearly proved fatal. Upon it being suggested later that the operation be repeated, the patient, a young man, said if we and his family insisted to avoid it he would commit suicide. Dr. W. S. Armstrong was then called in with me. He suggested an exclusively milk diet, gradual reduction of opium, and Rockbridge (Va.) iron and alum water. Immediate improvement set in, and in ten days so great was the constipation that oil and enemas had to be resorted to to procure an evacuation from the bowels. Since that time I have treated several cases equally as bad, with same happy result, using same diet and medicines, for of all agents for this disease, I consider the Rockbridge alum and iron water the first. It is equally efficacious in chronic diarrhœa, as my records attest. While I have not used it in chronic hemorrhages, bronchial catarrhs, mucous *pro fluviæ* generally, analogy and the chemistry of the water would suggest it as a valuable agent. Failure will result if you attempt to get medicinal effect by using the iron and alum mass as sold in the drug stores. It is the solids contained in the water after evaporation and of course should and probably does contain all the salts, etc. Directions accompany each package as to how much water is to be added to make the natural water.

Here, as is often the case, we can analyze but not synthatize.

